# 

**Published:** 1900-01-06

**Authors:** 


					THE HOSPITAL. " The standard of highest purity."?Lancet. January 6tii, 1900.
" CADBURY'S Ff8* |f J* CADBURY'S
COGOA % mm GOCOA
ABSOLUTELY PURE. NO DRUGS OR ALKALIE8 USED.
mkmm;
No. 693. 1 Vol. XXVII. Saturday,
Jan. 6, 1900.
bTp?222 J PRICE TWOPENCE.

				

